# Emergent Bacteria in Cystic Fibrosis: *In Vitro* Biofilm Formation and Resilience under Variable Oxygen Conditions

**DOI:** 10.1155/2014/678301

**Published:** 2014-04-29

**Authors:** Susana P. Lopes, Nuno F. Azevedo, Maria O. Pereira

**Affiliations:** ^1^Institute for Biotechnology and Bioengineering (IBB), Centre for Biological Engineering, Universidade do Minho, Campus de Gualtar, 4710-057 Braga, Portugal; ^2^LEPAE, Department of Chemical Engineering, Faculty of Engineering, University of Porto, 4200-465 Porto, Portugal

## Abstract

Concurrent to conventional bacterial pathogens, unusual microbes are emerging from cystic fibrosis (CF) airways. Nonetheless, little is known about the contribution of these newly microbes to the resilience of CF-associated biofilms, particularly under variable-oxygen concentrations that are known to occur *in vivo* in the mucus of CF patients. Two CF-emergent bacterial species, *Inquilinus limosus* and *Dolosigranulum pigrum*, and the major pathogen *Pseudomonas aeruginosa* were studied in terms of biofilm development and antibiotic susceptibilities under *in vitro* atmospheres with different oxygen availabilities. All species were able to develop *in vitro* biofilms under different oxygen-available environments, with *D. pigrum* accumulating high amounts of biomass and respiratory activities. When established, biofilms were of difficult eradication, with antibiotics losing their effectiveness in comparison with the corresponding planktonic populations. Surprisingly, biofilms of each emergent organism displayed multidrug resistance under aerobic environments, enduring even in low-oxygen atmospheres. This study suggests a potential prospect on the impact of nonconventional organisms *I. limosus* and *D. pigrum* on CF lung infections, demonstrating capacity to adapt to biofilm mode of life under restricted-oxygen atmospheres resembling CF airways, which may ultimately endanger the efficacy of currently used antibiotic regimens.

## 1. Introduction

Heterogeneous microenvironments are known to occur within the airways of cystic fibrosis (CF) patients. The presence of antibiotics, nutrients (e.g., products of inflammatory cell death, such as DNA and actin polymers), as well as zones of distinct oxygen tensions, promotes favorable conditions for bacterial colonization [1], contributing to the multispecies nature of the infection [2–4]. Arguably,* P. aeruginosa *is considered the prevailing pathogen in CF airways, leading to severe infections that eventually afflict almost all CF patients and are responsible for the progressive lung injury [5].* P. aeruginosa *is able to grow in the stagnant mucus that is built up on lung airway epithelia, surviving and adapting into the anaerobic layers [6, 7] throughout a series of genetic and phenotypic changes, namely, the formation of biofilms of difficult eradication [8].

However, it is now known that CF is not a monomicrobial disease, supporting a wide variety of many uncommon species [9].* Inquilinus limosus* and* Dolosigranulum pigrum *are two emergent bacteria recently reported in expectorated CF patients [10].* I. limosus* is an aerobic gram-negative bacillus from the subdivision of *α*-Proteobacteria [11] and has been pointed as a potential threat for CF patients, mainly due to the mucoid physiology, the multidrug resistance pattern, and the ability to persist in the respiratory tract [12].* D. pigrum* is a facultative anaerobe that was firstly described for nearly two decades after being isolated from human sources [13]. The upper respiratory tract is thought to be the natural habitat of* D. pigrum *[14], being associated with ventilator-associated pneumonia, nosocomial pneumonia, and septicemia [15, 16]. As for* I. limosus*, the full pathogenic potential of* D. pigrum* in CF remains unclear, with little information available about its physiological and phenotypic features under low-oxygen conditions and even less about its role in the increasing overall resistance to antibiotic therapy of CF traditional pathogens. Thus, evaluating the fitness of uncommon bacteria in* in vitro* conditions resembling CF airways may give major insights into their contributions for CF, being a starting point to determine their potential for pathogenicity.

As such, this study aimed to investigate the ability of* I. limosus* and* D. pigrum* to develop biofilms and to resist against several antibiotics under* in vitro* oxygen environments (aerobiosis, microaerophilia and anaerobiosis) broadly representing the microenvironments found in the CF airway mucus. Results were compared with the performance for* P. aeruginosa*.

## 2. Material and Methods

### 2.1. Bacterial Strains and Culture Conditions

*P. aeruginosa *(wild-type strain UCBPP-PA14),* I. limosus* (strain M53, isolated from CF sputum, kindly provided by Dr. Michael Surette, University of Calgary, AB, Canada), and* D. pigrum* (CIP 104051T, Institute Pasteur Collection, Paris, France) were used throughout this work. All strains were stored at −70 ± 2°C in tryptic soy broth (TSB, Liofilchem, Italy) supplemented with glycerol. Prior to each assay, bacteria were subcultured twice from frozen stock preparations onto TSB supplemented with 1.2% (wt/vol) agar plates and incubated aerobically at 37°C for 24–48 h. Pure liquid cultures of* P. aeruginosa *and* I. limosus *were made in TSB whereas* D. pigrum* was maintained in brain heart infusion broth (BHI, Liofilchem, Italy). Tryptic soy agar (i.e., TSB supplemented with the agar, as described before) was used as culture medium for CFU countings.

Unless otherwise stated, all rinse steps were performed either by using 0.9% (wt/vol) saline solution (NaCl; J. T. Baker, Deventer, The Netherlands) or distilled sterile water.

### 2.2. Biofilm Growth* In Vitro *(Biofilm Growth Curves)

Biofilms of each species were formed as previously described [17], with some modifications. Shortly, cell suspensions standardized with 1.0 McFarland standards were diluted in the respective broth medium to achieve ~10^7^ cells/mL, dispensed in 96-well microtiter plates, and incubated at 37°C, 120 rpm, under aerobic, microaerophilic, and anaerobic environments. For aerobic assays, microtiter plates were placed in a standard incubator (n-biotek, Model NB-205Q, Korea). Microaerophilic biofilms were formed in a microaerophilic incubator (Thermo Scientific, Forma 311, USA), previously calibrated with 5% (vol/vol) CO_2_. The anaerobic atmosphere was created by sealing the plates containing the cell suspensions in plastic boxes with AnaeroGen (Oxoid Limited, Hampshire, England). Biofilm growth (cultivable cells) was analyzed every 2 h, until 24 h. At each time point (each 2 h), the content of the microtiter plates (planktonic fraction) was discarded and the wells were washed once with sterile 0.9% (wt/vol) saline solution. Biofilms were then detached by sonication using an ultrasound bath (Sonicor model SC-52, UK) and cultivable counts were determined on TSA plates, after aerobic incubation at 37°C. For each time point, the old anaerobic generation bag was replaced by a new one, in order to warrant anaerobic conditions inside the plastic box. Biofilm growth curves allowed determining the time needed for biofilms of each species to achieve the threshold cell concentration interval of 2 × 10^5^ to 2 × 10^6^ CFU/cm2. Three independent assays were performed for each species and condition.

### 2.3. Biofilm Analysis

Microtiter plates containing the biofilms within the threshold cell concentration of 2 × 10^5^ to 2 × 10^6^ CFU/cm^2^ (determined by biofilm growth curves) were removed from the different incubators, the wells were washed with saline solution (200 *μ*L per well) after discarding planktonic fraction, and the wells-attached bacteria were analysed in terms of biomass and respiratory activity. Wells containing only broth medium were used as negative controls. All tests were run in three independent assays.

*Biomass. *Biomass was quantified by the crystal violet (CV) staining method, using the procedure previously outlined [17], with minor modifications. Briefly, wells allowed air drying for 10 min after washing. Attached bacteria were then fixed with methanol (Fisher Scientific, Leicestershire, UK) for 15 min and stained with 1% (vol/vol) CV (Merck, Germany) for 1 min. The excess stain was removed by aspirating the content of each well and washed twice with double-distilled water. Lastly, wells were decolorized with pure methanol and the optical density of the obtained solution was measured at 550 nm (OD_550 nm_) using a microtiter plate reader (Model Sunrise-basic Tecan, Austria). 

*Metabolic Activity.* The metabolic activity of biofilms was measured using the 2,3-bis(2-methoxy-4-nitro-5-sulfophenyl)-2H-tetrazolium-5-carboxanilide sodium salt (XTT) colorimetric method described by Stevens and Olsen [18], with some modifications. Basically, after biofilm growth and washing procedures, 200 *μ*L of a combined solution of XTT (Sigma) and phenazine methosulfate (PMS) (Sigma) in a ratio of 15 : 1 was applied to adhered cells and plates were incubated at 37°C for 3 h in the dark. Biofilm activity was determined through the measurement of the optical density at 490 nm (OD_490 nm_) in each well, using a microtiter plate reader.

### 2.4. Antimicrobial Agents

Stock solutions of eight antibiotics, tobramycin (Merck, USA), gentamicin, levofloxacin, ciprofloxacin, clindamycin, cefotaxime, chloramphenicol, and rifampicin (all from Sigma-Aldrich), were prepared at 5120 mg/L and stored according to the manufacturers' instructions.

### 2.5. Planktonic and Biofilm Antibiotic Susceptibilities

The minimum inhibitory concentrations (MICs) and the minimum biofilm eradication concentrations (MBECs) were determined by adapting the protocol described for the Calgary Biofilm Device (CBD) [19] to the standard microtiter plates. Basically, weakly and nonadherent bacteria from biofilms grown in the microtiter plate wells were removed by washing the wells with saline solution. Attached bacteria (biofilms) were then exposed to increasing 2-fold antibiotic concentrations prepared in cation-adjusted Mueller-Hinton broth (CAMHB) (for* P. aeruginosa* and* I. limosus*) or in CAMHB supplemented with 5% (vol/vol) sheep blood for* D. pigrum *cultures. Microtiter plates were incubated at 37°C for 24 h, under aerobic, microaerophilic, and anaerobic environments, as described for biofilm formation. Planktonic fractions delivered from treated biofilms were transferred to new microtiter plates and the MICs were obtained by reading the optical density at 650 nm (OD_650_) for clear wells (OD_650_ < 0.1). The MICs for* D. pigrum* were determined by visual observation of the turbidity gradient on the challenge plate. The remaining biofilms attached to the microtiter plate wells were rinsed twice with saline solution and disrupted by sonication (by ultrasound bath), into the appropriate broth supplemented with 1% (vol/vol) tween 20 (200 *μ*L per well). Disrupted biofilms were then plated onto TSA and incubated for 37°C (24–48 h). The MBECs were determined by CFU counting, being the lowest antibiotic concentration that could eradicate at least 99% of biofilm-encased cells.

### 2.6. Statistical Analysis

Data were analyzed using the Prism software package (GraphPad Software version 5.0 for Macintosh). Biofilm mass and activity were compared by one-way analysis of variance (ANOVA) and applying the Bonferroni posttest to subsequently compare pairs of columns. Results were considered statistically significant when *P* < 0.05. Raw data obtained for this paper is available at http://www.biofomics.org/ [20].

## 3. Results and Discussion

This study aimed to appraise the adaptation to biofilm mode of growth of* I. limosus* and* D. pigrum* and determine their respective antibiotic susceptibilities in variable-oxygen atmospheres resembling CF airways.* I. limosus* and* D. pigrum* are two recently recovered species from the secretions of CF patients [10, 11]. Unlike* P. aeruginosa*, which has been extensively studied in such environments [21–23], no reports were found to evaluate the performance of such emergent bacteria while associated with biofilms in the oxygen conditions found in* in vivo* CF airways. The literature available so far has only demonstrated their resistance patterns under standard environments and in planktonic cultures [12, 14], failing to consider the role of biofilms, of anaerobiosis, and of polymicrobial infections in CF.

**Figure 1:**
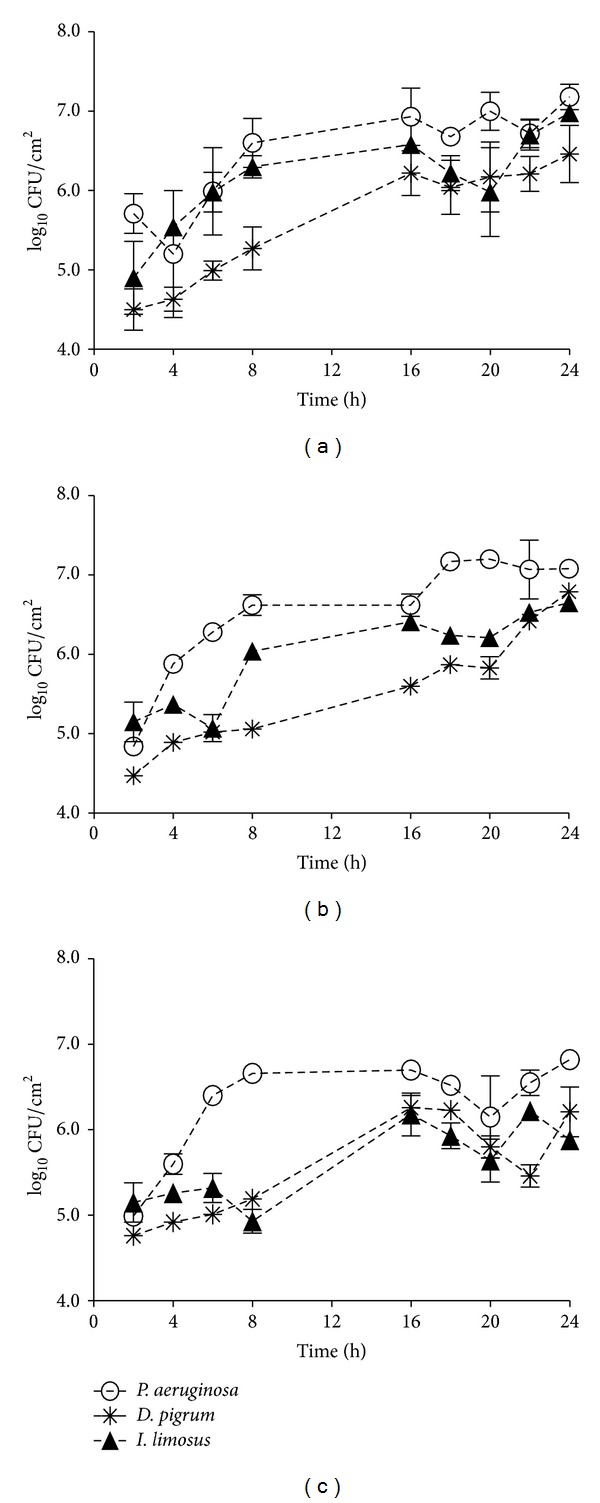
Growth curves obtained for* P. aeruginosa, D. pigrum, *and* I. limosus* single-biofilms growing under aerobic (a), microaerophilic (b), and anaerobic (c) environments. The means ± standard deviations for three independent assays are illustrated.

**Figure 2:**
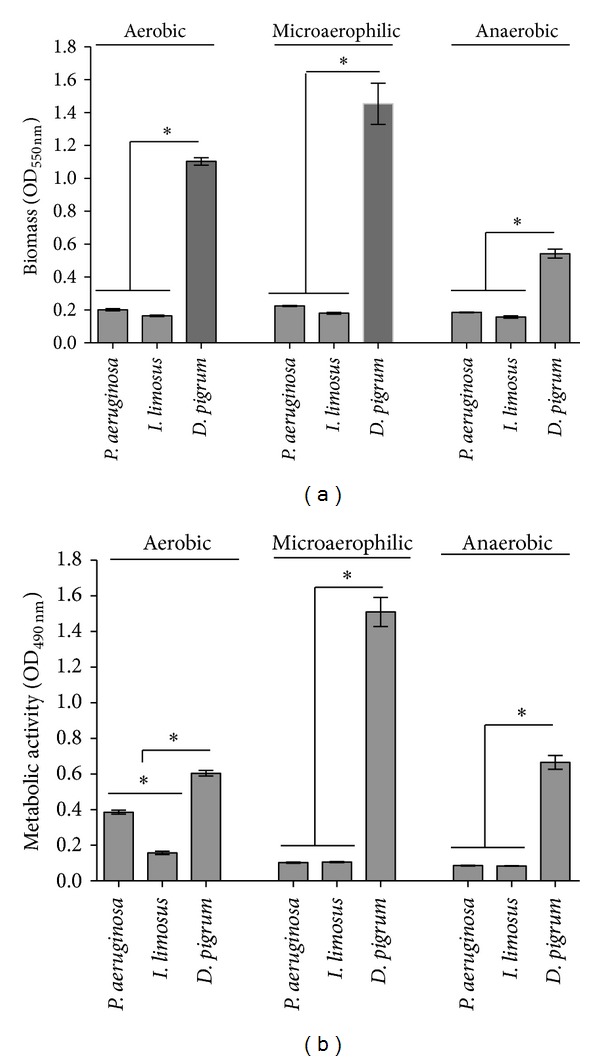
Biomass (a) and metabolic activity (b) obtained for single-biofilms formed by* P. aeruginosa*,* I. limosus,* and* D. pigrum *developed under aerobic, microaerophilic, and anaerobic environments. The means ± standard deviations for three independent assays are illustrated. *P < 0.05 (one-way ANOVA, Bonferroni's posttest).

Results revealed that, as for* P. aeruginosa*,* I. limosus *and* D. pigrum* are able to adapt and survive in variable-oxygen atmospheres growing as biofilms (Figure 1), showing high specific growth rates (in orders or magnitude ranging between 10^4^ and 10^5^ cells/cm^2^/h). Indeed, the biofilm mode of growth has been shown to play an important role in the evolution of bacterial phenotypic diversification, which is commonly associated with specialized adaptation to the different compartments in the CF airways [24, 25]. A good example has been shown for* P. aeruginosa*, which can survive for long periods of time under the challenging environment in CF under these circumstances. The adaptation is clearly understandable for* D. pigrum* that, as a facultative anaerobe, may more easily thrive under low-oxygen conditions.* Inquilinus* genus was initially characterized as aerobic [11], but the survival of* I. limosus* isolate under variable-oxygen tensions in this study might suggest that not all isolates are necessarily aerobic and may persist under different oxygen concentration zones within the mucus. Accordingly, Chiron and colleagues had already detected the presence of isolates able to survive under both conditions (aerobic and anaerobic) [12], leading to believe that different strains may be recovered from different compartments within the mucus. Additionally, Chiron et al. detected the presence of strains with nonmucoid and mucoid phenotypes, suggesting that* Inquilinus* might be able to undergo a switch to a mucoid phenotype, leading to biofilm formation and chronic colonization in CF airways. In this study, we observe that the CF isolate* I. limosus* M53 presented a very mucoid physiology (see Figure S1 in Supplementary Material available online at http://dx.doi.org/10.1155/2014/678301), which likely contributed to the slow growth under* in vitro* conditions and consequently to the limited biomass and metabolic activity under the different oxygen concentrations (Figure 2). For a similar number of CFUs per area of biofilm (ranging between 2 × 10^5^ and 2 × 10^6^ CFU/cm^2^),* D. pigrum *presented significant values of biomass and respiratory activity compared with the other species (*P* < 0.0001) (Figure 2). Originally, this observation was supposed to be attributed to a possibly higher matrix content and cellular activity of* D. pigrum* biofilm-encased cells. However, after determining the biochemical composition of biofilms (Figure S2), it was observed that the amount of matrix produced by* D. pigrum* was not significantly higher than the matrix formed by* P. aeruginosa* and* I. limosus*. This strongly suggests that* D. pigrum* biofilm could have more cells, but that those are in a viable (perceptible in Figure 2(b)) but nonculturable state. As in this study the number of CFUs before and after antibiotic application is compared, the fact of having more* D. pigrum* cells in the beginning of the experiment (but not CFUs) is unlikely to affect the final outcome concerning antibiotic efficacy assessment.* P. aeruginosa *and* I. limosus *biofilms presented similar biomass values in all environments, without displaying any significant discrepancies (*P* > 0.05). The same tendency was found for the metabolic activity of all biofilms (Figure 2(b)), with exception for aerobic conditions, where the classical species was more active than* I. limosus*.

Because antibiotherapy in CF patients generally targets only a limited number of microorganisms, in particular the major pathogen* P. aeruginosa* [26, 27], disregarding the impact of other microbes (including emergent) that are actually present, the antibiotic susceptibilities of* I. limosus* and* D. pigrum* were addressed in this study and compared with those obtained for* P. aeruginosa*. Planktonic cells seeded from treated biofilms served as the inoculum for MIC determinations, which better reflect infections on environmental settings, including the CF scenarios, where biofilms and planktonic cells form integrated parts of the microbial lifestyle. Conversely, the standard guidelines, where the antibiotics are applied to a standard planktonic culture, do not mirror well what occurs in* in vivo* CF infections. In this study, biofilms were highly recalcitrant to most antibiotics tested, whereas planktonic cells were in most cases fully susceptible (Table 1). In fact, these latter populations (in the planktonic state) seemed to be more sensitive to most antibiotics than biofilms. Whereas* I. limosus* planktonic populations did not show significant discrepancies in antibiotic susceptibilities under the different environments, the resistances of* D. pigrum* were noticeably declined under low- oxygen environments. Aminoglycosides (tobramycin and gentamicin) and fluoroquinolones (levofloxacin and ciprofloxacin) presented strong activity (MICs ≤ 2 mg/L) against planktonic* P. aeruginosa*, whereas for* I. limosus* only fluoroquinolones and rifampicin had a strong effect.

The MICs were not predictive of the MBECs. As expected, biofilms were notoriously more resistant to antibiotics than their planktonic counterparts, with MBEC values being, in general, too much higher than the MICs. There is increased evidence of enhanced tolerance associated with biofilms, allowing bacteria to survive, but not necessarily to grow, in the presence of antibiotic concentrations above their planktonic MIC [28]. The biofilm mode of growth is the main reason for the failure of antibiotic treatment to eradicate airway infection, allowing the bacteria to persist for decades in the CF lung [29]. Therefore, early treatment strategies are necessary to prevent or eradicate biofilm formation in the very early stages, and the maintenance of the intermittent colonization stages becomes crucial as well [30]. Otherwise, mutational resistance mechanisms arise, making management of the biofilm infection more difficult [29]. Biofilm tolerance is thought to be multifactorial, resulting from the oxygen and nutrient microscale heterogeneities within the biofilm; the protective barrier provided by the exopolysaccharide matrix, restricting or inactivating the penetration of antibiotics into the biofilm; the number and spatial distribution of bacterial cells within biofilms; the expression of biofilm-specific resistance genes; and the presence of “persisters,” that is, a subpopulation of microorganisms that differentiate into a dormant but protected state [31, 32].

Interestingly, an increase in antibiotic resistance was observed for* I. limosus* and* D. pigrum* biofilms. This extreme multidrug tolerance was endured even at oxygen-restricted conditions, with MBECs being higher than 1024 for at least 7 from a total of 8 antibiotics. Although the exact mechanism underlying the high tolerance to antibiotics is not clearly understood for both organisms, several reasons are pointed out. For* D. pigrum, *the high biomass achieved for these biofilms, associated with the high content of polysaccharides and proteins leading to a dense extracellular polymeric matrix (Figure S2), and the number of cells within the biofilms supposedly to be viable but noncultivable are suggested to significantly account for that antibiotic tolerance. In the case of* I. limosus*, the slimy character of bacterial colonies is clearly associated with the production of copious amounts of extracellular polymeric substances. It is reasonable that the mucoidy of* I. limosus* may constitute a physical barrier that limits the permeability of antibiotics and immobilizes/protects the biofilm-encased cells against killing. Also, cells buried in this biofilm presented reduced metabolic activity, making them less susceptible to antibiotics, which most (including aminoglycosides and fluoroquinolones, commonly used in CF treatment) are known to primarily target metabolically active biofilm subpopulations [33].

When extrapolated to the CF scenario, these results indicate that the presence of emergent bacteria in the CF airways community may lead to an ineffective antibiotherapy commonly applied to selected pathogens, worsening lung symptoms and contributing to the persistence of infection. Since biofilms have been considered an important pathogenic trait in CF chronic infections, persisting from years to decades without possible eradication [34] and assuming that* P. aeruginosa* can cause severe biofilm-associated infections, it is likely that the pathogenic potential of other species not known to exhibit pathogenic behavior may be measured by their ability to form biofilms [35–37]. Eventually, these atypical bacterial species may also interact with the traditional pathogens, increasing the overall resistance of the consortia [17]. As such, there is a need to fundamentally address these latest “holistic” approaches, since the microbe-microbe and microbe-host interplay within a given ecosystem may ultimately determine the properties and behaviours of the overall consortia [9].

In conclusion, although the most common CF pathogen is* P. aeruginosa*, this study has evidenced the pronounced ability of* I. limosus* and* D. pigrum* to grow and develop highly resilient biofilms under oxygen-limited atmospheres. The ability of emergent bacteria to persist under low-oxygen environments, resisting to antibiotic treatment, highlights their chance on the colonization and implication on CF lung infections. Thus, an adjustment to the actual therapeutic strategies, which are majorly focused on conventional pathogens, is necessary in face of the complex bacterial multiplicity and the highly resistant patterns associated with other than conventional organisms found in CF airways.

## Supplementary Material

Figure S1 shows a photograph of the mucoid physiology of I. limosus M53 strain used in this study, after growing onto TSA and incubated aerobically for 48 h. Figure S2 represents the content in protein and polysaccharides (in *μ*g per cm^2^) for the matrix and cells of the biofilms of *P. aeruginosa*, *I. limosus* and *D. pig rum* developed under aerobic, microaerophilic and anaerobic conditions.The total proteins content for biofilm matrix and cells was measured with the BCA Protein Assay Kit (Bicinchoninic Acid, Thermo Scientific, Rockford, IL, USA), using bovine serum albumin as the standard. The total polysaccharides content was estimated according to the phenol-sulphuric acid procedure of Dubois et al. (1956), by using glucose as the standard.

## Figures and Tables

**Table 1:** *In vitro* antibiotic susceptibilities of planktonic and biofilm populations formed by *P. aeruginosa *(PA), *I. limosus *(IL), and *D. pigrum *(DP) under aerobic, microaerophilic, and anaerobic environments.

Antibiotic	Aerobic			Microaerophilic			Anaerobic		
	PA	IL	DP	PA	IL	DP	PA	IL	DP
Tobramycin									
MIC^a^	≤2	64	512	≤2	128	64	≤2	64	64
MBEC	>1024	>1024	>1024	64	>1024	>1024	512	>1024	>1024
Gentamicin									
MIC	≤2	8	512	≤2	16	16	≤2	16	32
MBEC	>1024	>1024	>1024	128	>1024	>1024	1024	>1024	512
Levofloxacin									
MIC	≤2	≤2	512	≤2	≤2	16	≤2	≤2	16
MBEC	>1024	>1024	>1024	256	>1024	>1024	16	>1024	>1024
Ciprofloxacin									
MIC	≤2	≤2	>1024	≤2	≤2	16	≤2	≤2	32
MBEC	128	>1024	>1024	128	>1024	>1024	128	>1024	>1024
Clindamycin									
MIC	>1024	16	512	>1024	64	4	>1024	16	4
MBEC	>1024	>1024	>1024	>1024	>1024	>1024	>1024	>1024	>1024
Cefotaxime									
MIC	128	256	512	32	512	8	32	128	8
MBEC	>1024	>1024	>1024	>1024	>1024	>1024	>1024	>1024	>1024
Chloramphenicol									
MIC	512	256	256	64	128	8	64	128	16
MBEC	>1024	>1024	>1024	>1024	>1024	>1024	>1024	>1024	>1024
Rifampicin									
MIC	512	8	1024	>1024	≤2	≤2	>1024	≤2	≤2
MBEC	>1024	256	512	>1024	>1024	>1024	>1024	>1024	64

^a^MIC and MBEC values are expressed in mg/L.
