# Supplementary material for: Emergent Bacteria in Cystic Fibrosis: In Vitro Biofilm Formation and Resilience under Variable Oxygen Conditions
Source: Biomed Res Int. 2014 Apr 29;2014:678301. doi: 10.1155/2014/678301 (PMC4020565; doi:10.1155/2014/678301)
Supplement: Supplementary file 1 — Figure S1 shows a photograph of the mucoid physiology of I. limosus M53 strain used in this study, after growing onto TSA and incubated aerobically for 48 h. Figure S2 represents the content in protein and polysaccharides (in μg per cm2) for the matrix and cells of the biofilms of P. aeruginosa, I. limosus and D. pig rum developed under aerobic, microaerophilic and anaerobic conditions.The total proteins content for biofilm matrix and cells was measured with the BCA Protein Assay Kit (Bicinchoninic Acid, Thermo Scientific, Rockford, IL, USA), using bovine serum albumin as the standard. The total polysaccharides content was estimated according to the phenol-sulphuric acid procedure of Dubois et al. (1956), by using glucose as the standard. [file 678301.f1.zip › Figure_S2_Editable_2738_904427.docx]

**Figure S2** Protein (clean bars) and polysaccharides (streaked bars) content obtained from the matrix (white bars) and cells (grey bars) of single biofilms formed by *P. aeruginosa* (PA), *I. limosus* (IL) and *D. pigrum* (DP) developed under aerobic, microaerophilic and anaerobic environments. The means ± SDs for three independent assays are illustrated.
